# Activation of T Lymphocytes in Response to Persistent Bacterial Infection: Induction of CD11b and of Toll-Like Receptors on T Cells

**DOI:** 10.4061/2010/526740

**Published:** 2010-04-22

**Authors:** Dimitra Kotsougiani, Marco Pioch, Birgit Prior, Volkmar Heppert, G. Maria Hänsch, Christof Wagner

**Affiliations:** ^1^Institut für Immunologie, Universität Heidelberg, 69120 Heidelberg, Germany; ^2^Klinik für Unfallchirurgie und Orthopädie, Berufsgenossenschaftliche Unfallklinik, 67071 Ludwigshafen, Germany; ^3^Abteilung für Septische Chirurgie, Knochen-, Gelenk- und Protheseninfektionen, Berufsgenossenschaftliche Unfallklinik, 67071 Ludwigshafen, Germany

## Abstract

T cell activation is invariably associated with virus infections, but activation of T cells is also noted, for example, in patients with persistent bacterial infections with intracellular pathogens or localised bacterial biofilms. The latter is characterised by a destructive inflammatory process. Massive infiltration of leukocytes, predominantly of polymorphonuclear neutrophils (PMNs) and of T lymphocytes, is seen. While PMN influx into sites of bacterial infection is in line with their role as “first-line defence” a role of T cells in bacterial infection has not yet been delineated. We now found evidence for activation and expansion of peripheral blood T cells and an upregulation of Toll-like receptors 1, 2, and 4 on small portions of T cells. T cells recovered from the infected site were terminally differentiated and produced interferon gamma, a cytokine known to enhance functions of phagocytic cells, leading to the conclusion that infiltrated T cells support the local immuner defence.

## 1. Introduction

Activation and expansion of T lymphocytes is invariably associated with the immune response to virus infection, and the clearance of virus-infected cells. Activation of T cells, however, is also seen in bacterial infection, particularly in those caused by intracellular bacteria [1, 2] and—as we showed recently—in patients with implant-associated osteomyelitis, a prototype of a biofilm infection [3, 4]. 

Bacterial biofilms are increasingly recognised as the cause for persistent and destructive inflammatory processes [5, 6]. According to Donlan and Costerton [7], biofilms are defined as “microbial derived sessile communities characterised by cells that are irreversibly attached to a substratum or interface or to each other, embedded in a matrix of extracellular polymeric substances that they have produced, and exhibit an altered phenotype with respect to growth rate and gene transcription.” It is generally assumed that bacteria in biofilms escape the host defence. In vitro data suggest that bacteria in biofilms as not as susceptible to the phagocytic effector functions as their planktonic living counterparts [8, 9]; there is, however, no doubt that biofilms are not entirely protected [10] and that biofilm infection elicit an activation of the immune response with an infiltration into the infected site of leukocytes, predominantly of polymorphonuclear neutrophils (PMNs) and T lymphocytes [3, 4, 11–13]. 

While the participation of PMN in the defence against bacterial infection and in the acute inflammatory response is well understood, a role for T cells has not yet been delineated, nor is it known how T cells recognise bacteria. In that context, the aim of the present study was to analyse T cells of patients with persistent bacterial infections with regard to expression of activation-associated receptors on T cells, particularly of Toll-like receptors (TLRs) on. TLRs recognise conserved microbial structures including lipopolysaccharides (LPSs), lipoteichoic acid (LTA), lipopeptides, or bacterial DNA and RNA. As the so-called “pattern recognition receptors” TLRs are primarily studied on cells of the innate immune response; however, TLR are also detected on T cells, and a modulatory function of the specific, adaptive immune response via TLRs is presumed [14–17]. We focussed on TLR1, TLR2, and TLR4, because these receptors alone or in combination recognise the bacterial products LTA or LPS which might be present at the site of infection. Moreover, to further characterise T cells we determined surface receptors known to be associated with activation, such as CD11b and CD57, as well as production of interferon gamma, a cytokine known for its capacity to activate phagocytic cells.

## 2. Patients, Material, and Methods

### 2.1. Patients and Donors

After having obtained informed consent and after approval by the local ethic committee, 38 patients with implant-associated infections requiring surgery for removal of the implant or the endoprosthesis were recruited into the study between 1/2004 and 5/2007. The diagnosis was based on the medical history, the clinical examination, and X-rays. In the majority of patients, the leukocyte count was within the normal range (5 to 10 × 10^3^/ *μ*L); leukocytosis was seen in 8 patients. The C-reactive protein (CRP) serum concentrations were enhanced in 34 patients. The patients differed with regard to age (23 and 84 years), sex, localisation of the implant/endoprosthesis, and the interval between the primary implantation and the development of the clinically apparent osteomyelitis (2 months to 5 years). Intraoperatively, in all patients a prominent leukocyte infiltration and pus formation was observed, confirming the diagnosis of osteomyelitis. From all patients swabs were taken intraoperatively for the identification of bacteria with the following results: *Staphylococcus aureus *(*n* = 12); *S. epidermidis *(*n* = 7), Streptococci (*n* = 3), *E. coli *(*n* = 1), and propionibacter (*n* = 1). One patient presented with *Candida albicans*, and in 12 patients no bacteria were identified. The failure to detect bacteria by conventional diagnostic methods, however, does not rule out infection, since it is known that in implant-associated infection bacteria tend to form biofilms. Due to this specialised life-form, the bacteria do not grow as rapidly as their free-swimming planktonic counterparts and therefore may escape detection. 

From the patients, venous blood (5 mL) was taken immediately prior to surgery. For comparison, blood of healthy donors (*n* = 20) was taken (informed consent was obtained and the institutional guidelines were observed). Because some of the parameters, particularly expression of CD28 and CD57 increased with age, the donors were age matched. The cytofluorometric studies (see below) were carried out within 3 hours after blood withdrawal. Of note, for technical reasons, specifially the limited number of cells, not all experiments were carried with cells of all patients. The actual number of patients for one particularly parameter is given in the respective figure legend.

### 2.2. Recovery of the Leukocytes from the Infected Site

During surgery the tissue surrounding the infected implant/endoprosthesis was rinsed with 50 to 100 mL of sterile pyrogene-free 0.9% saline using a 50 mL syringe. The so-called “lavage” was collected in sterile tubes containing heparin (2500 IU per 50 mL) and sodium azide (final concentration 0.1%). The samples were processed within 3 hours. The cells were collected by centrifugation (10 min; 1500 rpm), washed with sterile phosphate buffered saline (PBS) containing 0.1% bovine serum albumin, and subjected to cytofluorometry. Between 5 × 10^5^ to 10^7^ leukocytes could be harvested.

### 2.3. Cytofluorometry

Labelling of cells was performed using the following antibodies: FITC-labelled antibody to CD57 (Becton Dickinson Biosciences Pharmingen; Heidelberg, Germany); allophycocyanin (APC)-labelled antibody to CD4 (Becton Dickinson); APC-labelled isotypic mouse IgG1 (Becton Dickinson); PE-labelled antibody to CD28 (Clone 28.1; DakoCytomation; Glostrup, Denmark); FITC-labelled antibody to CD11b (Biozol); FITC-labelled isotypic mouse IgG1 (Immunotech; Marseille, France); APC-labelled antibody to CD8 (BD Biosciences Pharmingen); PE-labelled antibody to CD8 (Becton Dickinson); CyChrome-labelled antibody to CD56 (BD Biosciences Pharmingen); CyChrome-labelled isotypic mouse IgG1 (Becton Dickinson); phycoerythrin (PE)-labelled anti-mouse IgG2a (Biozol; Eching, Germany); PE-labelled antibody to CD4 (Immunotech); FITC- and Phycoerythrin (PE)-labelled isotypic mouse IgG1/IgG2a (Immunotech). PE-conjugated antibodies to Toll-like receptors (eBioscience SanDiego, USA). The final concentration of antibodies varied between 1–20 *μ*g/mL of whole blood. Following incubation with the respective antibodies (20 minutes, room temperature), erythrocytes were lysed using FACS-lyzing solution provided by Becton Dickinson. Cells were analyzed by FACSCalibur and CellQuest software (Becton Dickinson). Results are expressed as percentage positive cells in the respective gate or quadrant. The markers were set according to the IgG isotype controls. Cells of the lavage were washed in PBS containing 1% bovine serum albumin and 0.1% sodium azide, adjusted to 10^6^/100 *μ*L and incubated with the respective antibodies (1 *μ*g/100 *μ*L). For intracellular staining, the cells were permeabilised using FACS/Perm solution and the protocol provided by Becton and Dickinson. The FITC-labelled antibody to interferon *γ* was purchased from Serotec (Düsseldorf, Germany).

**Statistics**

Data are presented as box and whiskers blots, with the box containing 50% of the measured values, the whiskers showing the highest and the lowest values, respectively, the horizontal bar the median values and the square the mean values. The differences between the mean values of the groups (cells from the patients versus cell of the healthy donors) were calculated using ANOVA.

## 3. Results

### 3.1. Evidence for the Activation of CD4+ T Cells in the Peripheral Blood of Patients with Implant-Associated Osteomyelitis

#### 3.1.1. Up-Regulation of CD11b and Loss of CD28

**Figure 1:**
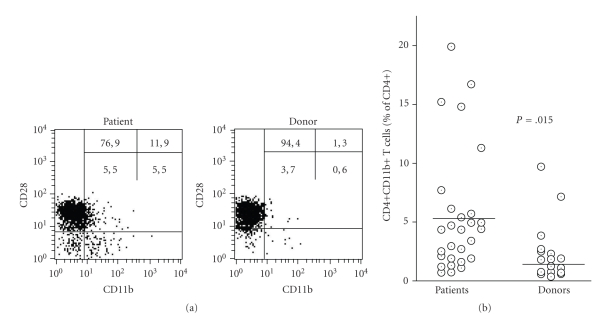
Expression of CD11b and CD28 on CD4+ T cells of patients with implant-associated osteomyelitis and of healthy donors: (a) by cytofluorometry expression of CD11b and CD28 were determined. The gate was set for CD4+ cells. With regard to CD11b and CD28 expressions, four populations were detected: CD28+CD11b-, CD28+CD11b+, CD28-CD11b-, and CD28-CD11b+ (an example for a patient and a donor is shown in (a) (The numbers give the percentage of CD4+ found in the respective quadrant.) (b) The percentage of CD4+CD11b+ in the patients (*n* = 28) and the donors (*n* = 18) is shown. The horizontal bar indicates the median value. The mean values for the patients (mean ± SD 5.6 ± 5.3%) differed significantly from that obtained for the donors (2.2 ± 2.4%) as calculated by ANOVA (*P* = .015).

Loss of CD28 and up-regulation of CD11b is associated with activation and expansion of T cells clones. To assess activation of T cells we determined these receptors on CD4+ T cells of patients with implant-associated osteomyelitis immediately before surgery. By cytofluorometry four cell populations could be determined: CD28+CD11b−, CD28+CD11b+, CD28−CD11b+, and CD28−CD11b−. T cells expressing CD11b were found in the majority of patients, and to a lesser extent in the healthy donors. Within the CD4+CD11b+ population, CD28− were prevalent (60 ± 17%) (example and summary of data in Figures 1(a) and 1(b)). That CD11b was preferentially associated with the CD28- cells is in line with the fact that CD4+CD28+CD11b+ represent an early transient phenotype, as opposed to the activated CD4+CD28−CD11b+ phenotype.

#### 3.1.2. Expression of Toll-Like Receptors

**Figure 2:**
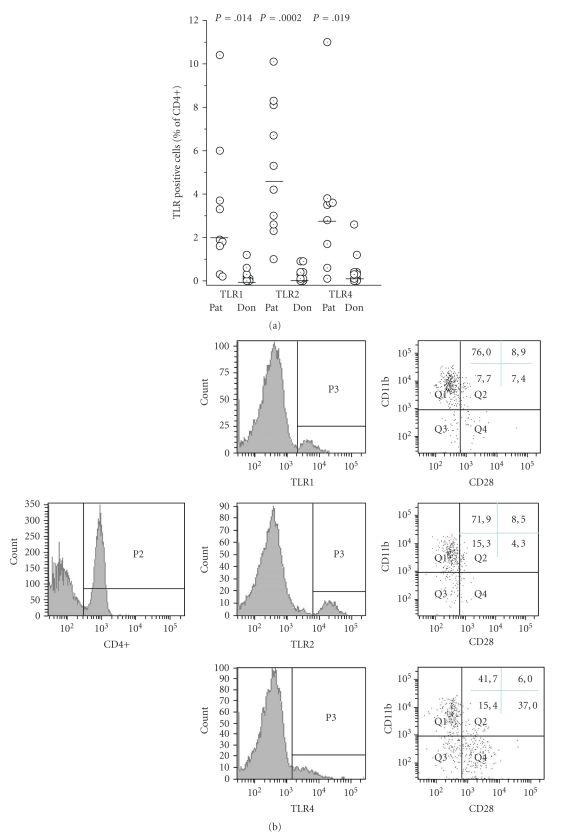

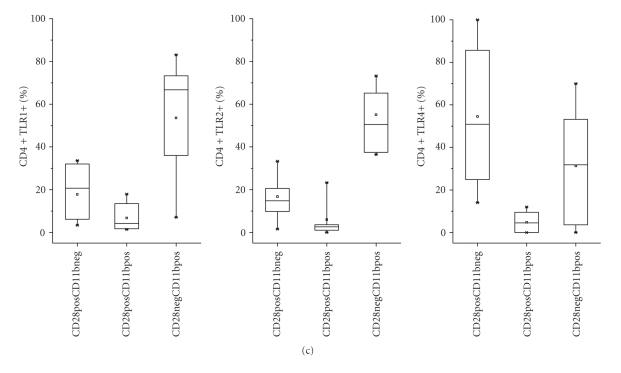
Expression of TLR on peripheral CD4+ T cells in patients with implant-associated osteomyelitis and healthy donors: (a) by cytofluorometry, expression of TLR1, TLR2, and TLR4 on CD4+ cells was determined. Each dot represents a patient or a donor, respectively. The horizontal bars represent the mean values. These were different between patients and donors as calculated by ANOVA. (b) When the gate (P2) was set for CD4+ cells, TLR positive cells could be identified (P3) (middle panel) and the expression of CD28 and CD11b on these cells could be determined (right panel). Four cell populations became apparent CD28-CD11b+ (Q1), CD28+CD11b+ (Q2), CD28-CD11b- (Q3), and CD28+CD11b- (Q4). The markers to determine positive or negative were set according to the isotype controls (not shown). The numbers refer to the percentage of cells in the respective quadrant and are calculated as per cent of TLR positive cells. In (b) an example for a single patient is shown, in (c) data of 10 patients and 9 donors are summarised in box and whiskers plots, with the horizontal bar showing the median, and the dot showing the mean values. (Again, markers to defines positive or negative were set according to the appropriate isotype control.)

In the peripheral blood of healthy donors, less than 1% of T cells express TLR1, TLR2, or TLR4. In the majority of patients with implant-associated osteomyelitis, however, T cells expressing TLR1, TLR2 or TLR4 were detected. A preferential association of TLR1 and TLR2 expressions with CD4+CD28−CD11b+ cells was seen, compatible with the fact these cells represent an activated phenotype (data summarised in Figure 2).

#### 3.1.3. Association of T Cell Activation with the Infection

**Figure 3:**
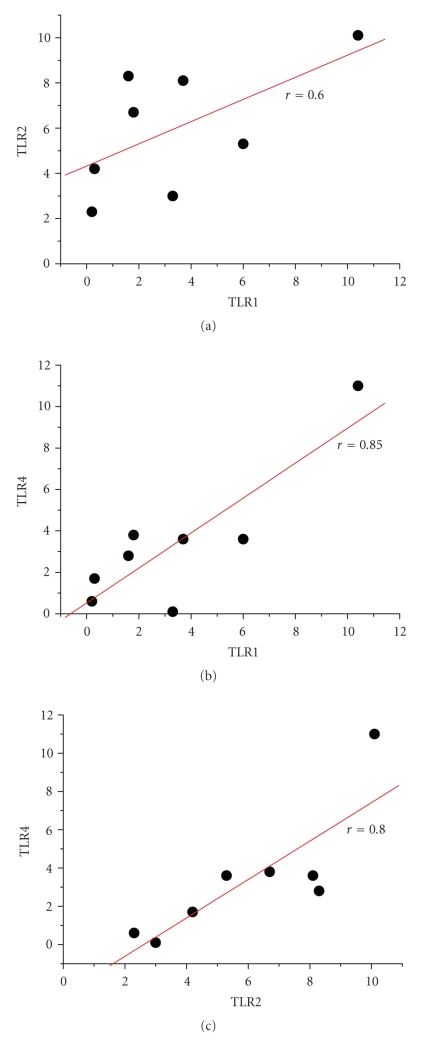
Assessment of linear correlations of TLR expression: TLR expression on CD4+ cells (values represent % TLR1, TLR2 or TLR4 positive CD4+ cells; each dot represents one patient) was plotted and by linear fit analysis the correlation was assessed. With *P* < .001, a weak correlation between TLR1 and TLR4 expression was seen, and between TLR2 and TLR4 expression, but not between TLR1 and TLR2.

**Figure 4:**
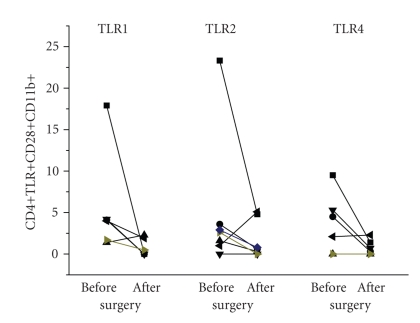
Presence of TLR positive cells in the peripheral blood of patients correlates with infection: CD4+CD28+CD11b+TLR1+, CD4+CD28+CD11b+TLR2, or CD4+CD28+CD11b+TLR4 was quantified by cytofluorometry at the day of surgery (before) or 6 to 8 days later (after), when the infection was resolved. Each symbol refers to an individual patient; the line connects the values before and after surgery.

We did not find correlations between the percentage of CD4+CD11b+ or TLR positive cells with the classical inflammatory parameters including serum CRP concentrations or leukocyte count; there was, however, a weak correlation between TLR1–TLR4, and TLR2–TLR4 expressions, but not between TLR1–TLR2 (Figure 3). From six patients we were able to test the peripheral blood cells after recovery (6 to 8 days after surgery). The percentage of CD4+CD11b+ and CD4+CD28− cells declined, as did the population of CD4+CD28+CD11b+TLR+ (Figure 4).

### 3.2. Analysis of Peripheral CD8+ Cells: Expression of CD11b, CD28, and TLR

**Figure 5:**
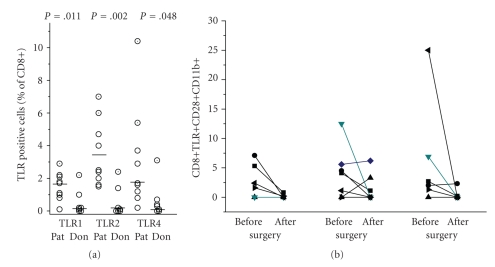
Expression of TLR on peripheral CD8+ cells in patients with implant-associated osteomyelitis and in healthy donors: (a) by cytofluorometry, expressions of TLR1, TLR2, and TLR4 on CD8+ cells was determined. Each dot represents a patient or a donor, respectively. The horizontal bars represent the median values. The mean values for TLR2 and TLR4 were different between patients and donors as calculated by ANOVA. (b) CD8+CD28+CD11b+ expressing TLR1, TLR2, or TLR4 were quantified by cytofluorometry at the day of surgery (before) or 6 to 8 days later (after) when the infection was resolved. Each symbol refers to an individual patient; the line connects the values before and after surgery.

When measuring CD28 and CD11b expression on CD8+ cells, no major differences between donors and patients were seen. On average, 28.5 ± 14.1% of the patients' CD8+ cells expressed CD11b versus 29.21 ± 14.7% of donors' cells, and 43.4 ± 22.9% were CD28 negative compared to 37.4 ± 20.3% in the donors (patients *n* = 28; donors *n* = 18). CD11b was predominantly seen on CD28− cells. Thus, when compared as a group no important differences were seen between patients and donors regarding CD28 and CD11b expressions. Small populations (3 to 5%) of CD8+ T cells expressing TLR2 and TLR4 were found in the majority of the patients, and to a lesser extent in healthy donors (Figure 5(a)). TLR expression was seen predominantly on CD11b+ cells. After recovery of the patients the percentage of CD8+CD28+CD11b+TLR+ cells declined, which was particularly obvious when compared within the same patient (Figure 5(b)).

### 3.3. Characterisation of the Infiltrated T Cells

**Figure 6:**
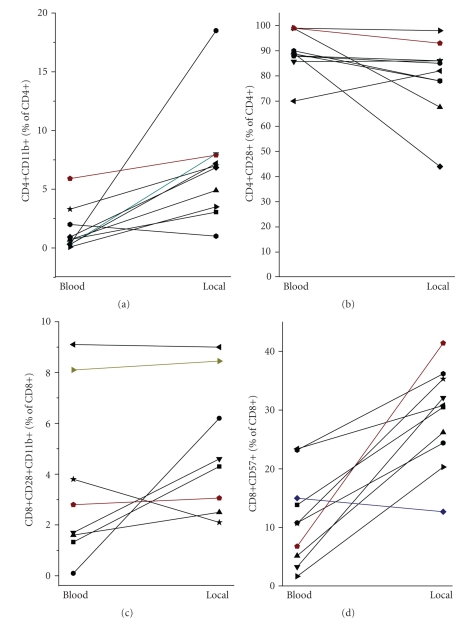
Characterisation of the infiltrated T cells: (a, b) compared to the peripheral blood, the cellular infiltrate (local) contained a higher percentage of CD4+CD11b+ cells, while CD28− was lost (each pair of symbols corresponds to one patient). (c) For CD8+ cells, a small increase of CD8+CD11b+ cells in the cellular infiltrate was seen in the majority of patients, and (d) a considerable in increase in CD8+ expressing CD57.

As reported before, CD4+ and CD8+ T cells were found at the infected site, with a shift towards CD8+ when compared to the peripheral blood of the same donor [3]. For CD4+ cells, an accumulation of CD11b+ and CD28− was seen, which was particularly noticable when compared within the same patient (Figures 6(a) and 6(b)). Again, for CD8+ the data were less convincing; in some patients the percentage of transiently activated CD8+CD28+CD11b+ phenotype was slightly higher at the infected site. In all, however, an accumulation CD8+CD57+ was seen. CD57 expression identifies terminally differentiated of CD8+ effector (Figures 6(c) and 6(d)). The expression pattern of the TLR was similar to that of the peripheral blood T cells, and an important accumulation at the infected site of TLR positive cells was not obvious.

### 3.4. Synthesis of Interferon *γ* by Peripheral and Infiltrated T Cells

**Figure 7:**
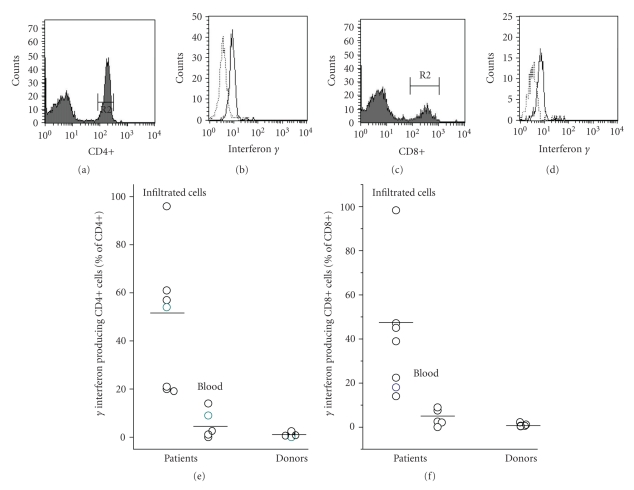
Synthesis of interferon *γ* by the infiltrated T cells. (a) The infiltrated T cells were recovered and cultured for 16 hours in the presence of brefeldin 1. The cells were permeabilised, and interferon *γ* was determined by cytofluorometry, as was CD4 and CD8. The gate was set for CD4+ or CD8+, respectively. Up to 90% of cells contained interferon *γ*. (b) Data of 7 patients are summarised. Shown are values obtained for the infiltrated T cells (local), for T cells of the peripheral blood of patients, and for healthy donors as well (differences of the mean values were calculated by ANOVA and found to be different with a *P* < .001).

To determine synthesis of interferon *γ*, the infiltrated leukocytes, and for comparison the cells in whole peripheral blood, were incubated for 4 to 16 hours in the presence of brefeldin 1 in order to allow accumulation of the cytokine in the cells. Interferon *γ* was then detected by cytofluorometry of the permeabilised cells. We found in the infiltrated T cells, but not in the peripheral blood T cells, interferon *γ* in up to 50% of the cells (Figure 7).

## 4. Discussion

Our data provide evidence for the activation and expansion of T cells in patients with persistent bacterial infection. As marker for activation we measured expression of CD11b, because this adhesion protein is associated with T cell activation [18–20] and thought to be a homing receptor of T lymphocytes for nonlymphoid tissue [21] such as an infected or inflamed site. In our patients, CD11b expression was particularly obvious on CD4+ T cells—quite in contrast to the situation in virus-infected patients, where CD8+ cells acquired CD11b [22]. CD11b expression was seen mainly on CD4+ cells that had lost CD28, in line with numerous in vitro studies which showed that activation and expansion of CD4+ cells is associated with a loss of CD28 [23]. That only a minor portion of CD4+ cells acquires CD11b is in line with an oligoclonal CD4+ T cell activation, as it is expected for an antigen-driven T cell activation. Of note, after recovery of the patients, the percentage of CD4+CD11b+ and CD4+CD28− declined, indicative of a causal relationship between infection and T cell activation. 

When analysing the CD8+ cells, the data were less clear-cut: no major differences between patients and donors were seen when comparing the percentage of CD8+CD28− cells or CD8+CD11b+ cells. The most likely explanation is that CD8+CD28− cells, in contrast to CD4+CD28− cells, persist following activation and form the pool of CD8+CD28−CD11b+ and CD8+CD28−CD11b− cells which increases with age and antigen contact [23–26]. Thus, smaller changes induced by an ongoing oligoclonal T cell activation might be obliterated. Only when compared within the same patient, CD8 activation became apparent: CD8+CD28+CD11b+ which represent a transiently activated T cell phenotype [3, 22] were found in small numbers in the patients, and disappeared within days after surgery (compare Figure 5). 

Another new aspect of our study concerned the expression of TLR. As pattern recognition receptors, TLR expression is predominantly studied on cells of the innate immune system. There is, however, also evidence for TLR expression on T cells. Moreover, numerous in vitro studies showed that engagement of TLR by their respective ligands modulate T cell activity, including up-regulation of interferon *γ* synthesis [14–17]. We found in the majority of patients CD4+ and CD8+ cells expressing of TLR, particularly TLR2, a receptor selective for lipoteichoic acid, a major component of the outer cell wall of Gram-positive bacteria. The association of the TLR with activated T cells, those having lost CD28 and expressing CD11b, suggests a role for TLR in the bacterial infection-induced T cell response. That preferentially TLR2 is up-regulated in line with the observation that in our patients infection with Gram-positive bacteria is prevalent.

Further evidence for T cell activation in the context of bacterial infection is their infiltration into the infected site. As reported before, 10 to 30% of the infiltrated leukocytes were identified as T cells. Both, CD4+ and CD8+ were found, with—as reported previously [3]—a predominance of CD8+ T effector cells. For CD4+ cells, there was evidence for an accumulation particularly of activated CD28− CD11b+ cells, in line with observation that CD11b might be a homing receptor directing activated T cells to nonlymphoid tissue [21]; whereas for CD8+ T cells we found an accumulation of cells expressing CD57, a receptor typical for terminally differentiated effector cells [27]. 

Given the fact that T cells are activated in the course of bacterial infection and infiltrate the affected site, the question arises whether or not they participate in the local immune defence. Terminally differentiated T cells are a rich source of numerous cytokines, and in our patients up to 90% of the infiltrated T cells synthesised interferon *γ*, apparent as an accumulation of the cytokine within the cells. Interferon *γ* is a potent activator of phagocytic cells. It prevents apoptosis of PMN and enhances their phagocytic and bactericidal activity [28, 29] and thus could support the local host defence. We have, so far, only indirect evidence for the function of interferon *γ* at the infected site. Infiltrated PMN express MHC class II antigens [30], which on PMN are exclusively up-regulated by T cell-derived cytokines or by direct contact with activated T cells [31, 32]. Whether infiltrated T cells indeed contribute to the local host defence remains to be shown; it is, however, an attractive hypothesis.

In conclusion our data provide evidence that in response to local, persistent bacterial infections T cells are activated and recruited to the inflammatory site. The up-regulation of Toll-like receptors could provide the T cells with means to sense the “danger” of bacterial infection.
